# Polidocanol foam sclerotherapy for patients with Goligher grade IV hemorrhoidal disease unwilling to undergo surgery: preliminary outcomes from a retrospective study with 6-month follow-up

**DOI:** 10.1007/s00384-026-05208-5

**Published:** 2026-07-28

**Authors:** Gianpiero Gravante, Veronica De Simone, Arcangelo Picciariello, Roberto Sorge, Pierpaolo Sileri, Gaetano Gallo

**Affiliations:** 1 Department of General Surgery, Azienda Sanitaria Locale ASL Lecce, Casarano, Italy; 2 Colorectal Surgery Unit, IRCCS San Raffaele Scientific Institute, Faculty of Medicine and Surgery, Vita-Salute University, Via Olgettina 60, 20132 Milan, Italy; 3 Department of Experimental Medicine, University of Salento, Lecce, Italy; 4 Department of Human Physiology, Laboratory of Biometry, University of Tor Vergata, Rome, Italy

**Keywords:** Pelvic floor diseases, Proctology, Hemorrhoids, Sclerotherapy

## Abstract

**Background:**

Polidocanol foam (PF) sclerotherapy has shown promising results in low- and intermediate-grade hemorrhoidal disease (HD), but evidence in advanced stages remains limited. This study aimed to evaluate patient-reported outcomes following PF sclerotherapy in patients with Goligher grade IV HD who declined surgical treatment.

**Methods:**

This retrospective analysis included patients with Goligher grade IV HD that declined hemorrhoidectomy after appropriate counseling and underwent 3% PF sclerotherapy between June and October 2025. Outcomes were assessed using symptom-focused subsets derived from the PROM-HISS and HDSS instruments at baseline, 7 days, 30 days, and 6 months. Primary outcome was the change in scores over time. Secondary outcomes included need for additional treatment, complications, and subsequent surgery.

**Results:**

Fifteen patients were included (mean age 58 ± 11 years; 53.3% male). At 6 months, 66.7% of patients requested no further hemorrhoidal treatment. Significant improvements were observed in both scores across all follow-up timepoints (*p* < 0.05). Pain, bleeding, and prolapse showed the most consistent reductions. One patient (6.7%) required hemorrhoidectomy within 30 days, four patients (26.7%) a second sclerotherapy session. No major complications were observed.

**Conclusions:**

In selected patients with Goligher grade IV HD who are unwilling or unfit to undergo surgery, PF sclerotherapy may be associated with short-term symptom improvement and may represent a symptom-oriented minimally invasive option for selected patients unwilling or unfit to undergo surgery.

## Introduction

Sclerotherapy for the treatment of hemorrhoidal disease (HD) has recently regained attention [1, 2]. The new formulation of existing products—particularly the foam preparation of 3% polidocanol [3, 4]—has improved the safety profile of the procedure and progressively replaced phenol oil as the preferred sclerosing agent [5]. Studies in patients with second-degree HD [6], as well as second- and third-degree HD in high-risk populations [7], have demonstrated notable efficacy [2], and preliminary but encouraging long-term outcomes have also been reported [6]. Furthermore, recurrences can be easily managed given that the procedure is office-based, minimally symptomatic, and readily repeatable as needed [8]. Sclerotherapy could therefore be considered a first-line treatment for lower Goligher grades [5].

In patients with higher Goligher grades, especially grade IV, current guidelines traditionally recommend more radical treatments—primarily excisional hemorrhoidectomy—due to the higher recurrence rates associated with less invasive approaches in this setting [9]. However, the well-known burden of postoperative pain following hemorrhoidectomy continues to represent a major deterrent, often delaying or discouraging patients from undergoing surgical treatment [10]. In real-world clinical practice, a substantial proportion of patients with advanced HD refuse surgery due to fear of pain, opting instead to pursue less invasive therapies despite their limited durability, or to tolerate persistent symptoms while avoiding hemorrhoidectomy. Continued conservative management often provides only partial and temporary symptom control in these patients, while office-based procedures such as rubber band ligation are generally unsuitable for fixed grade IV hemorrhoidal disease. Although oral flavonoids may provide temporary improvement in bleeding and discomfort, their long-term efficacy in advanced disease is limited, and their use may also be restricted in some patients receiving anticoagulant therapy. Consequently, a proportion of patients with advanced HD remain symptomatic despite conservative measures while continuing to refuse definitive surgical treatment.

In this context, a cohort of patients with Goligher grade IV HD voluntarily underwent polidocanol foam (PF) sclerotherapy for symptom control and were assessed using symptom-focused Patient-Reported Outcome Measures (PROMs). In the present study, we report the short-term clinical and patient-reported outcomes of PF sclerotherapy in this selected population.

## Materials and methods

This is a retrospective observational single-arm study of grade IV HD patients that opted for PF sclerotherapy instead of hemorrhoidectomy after adequate counseling. It was reported in accordance with the Strengthening the Reporting of Observational Studies in Epidemiology (STROBE) guidelines [11]. All procedures involving human participants were performed in accordance with the ethical standards of the institutional and/or national research committees and with the 1964 Declaration of Helsinki and its later amendments or comparable ethical standards.

Included patients were those with Goligher grade IV HD who presented between June 2025 and October 2025 and were treated with 3% PF sclerotherapy. Patients were initially evaluated during an outpatient visit, which included a structured symptom interview and a complete clinical assessment, and were informed about guideline-recommended treatment options for advanced-grade HD [9]. Patients who declined surgical treatment despite adequate counseling regarding the limitations and higher risk of recurrence associated with less invasive options were subsequently offered office-based 3% PF sclerotherapy as a palliative, symptom-oriented treatment at their explicit request. Exclusion criteria included Goligher grade I–III HD; acute thrombosed hemorrhoids; receipt of hemorrhoidal procedures other than 3% PF sclerotherapy; the presence of associated anorectal pathologies (including anal fistulas or abscesses, anal fissures, condylomas, tumors, rectocele, internal intussusception, rectal prolapse, ulcerative colitis, Crohn’s disease, or other anoperineal disorders); and a history of previous anorectal surgery.

### PROMs

Symptoms were assessed using the symptom domains of the Patient-Reported Outcome Measure–Hemorrhoidal Impact and Satisfaction Score (PROM-HISS) [12] and the Hemorrhoidal Disease Symptom Score (HDSS) with the Short Health Scale for Hemorrhoidal Disease (SHD-HD) [13]. The PROM-HISS score comprises three domains: (i) five HD symptoms (bleeding, pain, prolapse, soiling, and itching), each scored from 1 (least bothersome) to 5 (most bothersome); (ii) impact on daily activities (0–10); and (iii) treatment satisfaction (0–10) [12]. In the present study, only the symptom domain was included. The impact on daily activities domain was excluded due to a consistent floor effect, with all patients reporting minimal impact. The treatment satisfaction domain was not considered, as it is not applicable in the pre-treatment setting and therefore unsuitable for baseline comparison. The HDSS/SHD-HD instrument consists of two domains: the HDSS, which evaluates the same five symptoms based on frequency (0 = never to 4 = daily), and the SHD-HD, which assesses health-related quality of life through three items (score 1–7) and overall well-being (1 = very good to 7 = very poor) [13]. In this study, only the HDSS symptom domain was used, as a substantial proportion of patients experienced difficulty in completing the SHD-HD domain, limiting data reliability. These subsets have not undergone independent psychometric validation; therefore, the findings reflect the performance of the instruments as used in this study rather than their fully validated forms. However, the retained symptom components correspond to the core symptom domains originally developed and validated within the PROM-HISS and HDSS instruments, and were used in the present study as exploratory symptom-focused outcome measures rather than as independently validated composite scales. No modifications were made to the individual symptom items or their scoring system; only selected validated symptom domains of the original instruments were analyzed separately.

### Protocol

Patients were initially assessed through a detailed history and a proctological evaluation, including perineal and digital rectal examinations. When rectal bleeding or other symptoms suspicious for colorectal cancer were present, a complete colonoscopy or computed tomography colonography was requested (unless a recent examination was already available). Patients meeting the inclusion criteria were treated during a separate appointment: a flexible sigmoidoscopy was carried out to allow direct visualization of the internal HD and to perform the sclerotherapy. The procedure was performed using a Pentax EPK-i7010 processor and an E38-i10L colonoscope. With regard to sclerotherapy, patients were treated according to current consensus [3], in an outpatient setting and in the left lateral position. PF (Atoxysclerol® 3%, Gloria Med Pharma S.r.l., Menaggio, Italy) was prepared using the Tessari method [14], by mixing 2 mL of liquid polidocanol with 8 mL of air through two syringes connected by a three-way stopcock, obtaining a total volume of 10 mL of foam. Two milliliters of foam were injected into each pile during the retroflexed maneuver using a 23-G endoscopic single-use injection needle (Olympus, Hong Kong). All endoscopic injections, as well as clinical assessments, were performed by a well-trained colorectal surgeon (GGr) with more than 5000 endoscopic procedures.

Patients were reviewed in the outpatient clinic at 7, 30-days, and 6 months following treatment, at which time the HISS and HDSS scores were reassessed, and a physical examination of the perineum was performed. Data collected included basic demographics (age, sex), clinical scores, and Goligher grade (as reported by patients). All follow-up assessments were completed before database closure and statistical analysis. The primary outcome of this study was to describe PROMs results achieved with PF sclerotherapy in grade IV HD over time. Secondary outcomes included the request for definitive hemorrhoidectomy, additional PF treatments, complications. Treatment failure was defined a priori as persistence or incomplete resolution of hemorrhoidal symptoms after PF sclerotherapy, resulting in the need for additional treatment (repeat sclerotherapy or hemorrhoidectomy). Recurrence was defined a priori as reappearance of hemorrhoidal symptoms after documented complete initial symptom resolution during follow-up, resulting in the need for additional treatment.

### Statistical analysis

All data were inserted into an Excel database (Microsoft, Redmond, Washington, USA) and analyzed using the Statistical Package for the Social Sciences for Windows version 27.0 (SPSS, Chicago, Illinois, USA). Descriptive statistics are presented as mean ± standard deviation for continuous variables and frequencies for categorical variables. Normality assumptions were assessed using histograms and the Shapiro–Wilk test, that demonstrated normal distribution of the analyzed variables. Pearson’s correlation coefficients were calculated to assess the strength of association between each individual symptom domain and the total HISS and HDSS scores, in order to explore the relative contribution of individual symptom domains to the overall composite scores. Comparisons over time were performed using one-way repeated-measures ANOVA for continuous variables and chi-square test for categorical variables (Fisher’s exact test when expected cell counts were < 5). For repeated-measures ANOVA, the assumption of sphericity was assessed using Mauchly’s test. When violated, Greenhouse–Geisser correction would have been applied; in the present dataset, the assumption of sphericity was met for all analyzed variables. For multiple pairwise comparisons involving HISS and HDSS symptom domains across timepoints, *p* values were adjusted using the Benjamini–Hochberg false discovery rate correction. A *p* value < 0.05 was considered statistically significant.

## Results

From June to October 2025, 15 patients with Goligher grade IV hemorrhoidal disease underwent 3% PF sclerotherapy and were included in the analysis. All patients completed the 7-day and 30-day follow-up assessments. One patient subsequently underwent Milligan–Morgan hemorrhoidectomy within 30 days because of persistent symptoms and therefore did not complete the 6-month PROM assessment. The remaining 14 patients completed the 6-month follow-up evaluation.

**Fig. 1:**
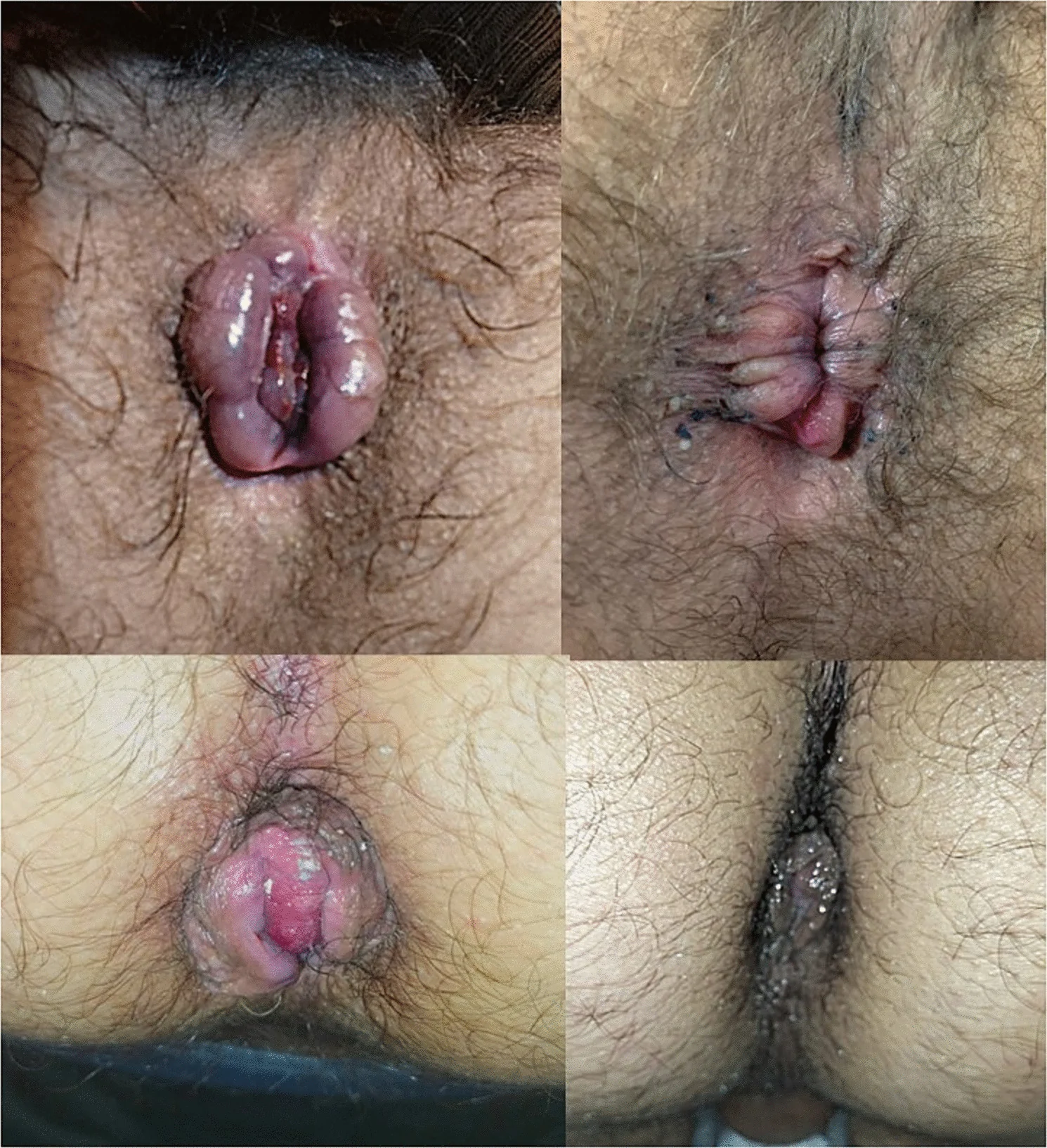
Representative clinical images of two patients with Goligher grade IV hemorrhoidal disease before treatment (left panel) and at 6-month follow-up after polidocanol foam sclerotherapy (right panel)

The mean age was 58 ± 11 years, and 8 patients (53.3%) were male. All patients reported symptoms lasting longer than one year; 14/15 (93.3%) had previously received conservative treatments, including oral flavonoids and topical therapies (e.g., steroid-based creams), without clinical benefit. At the 6-month follow-up, 10 patients (66.7%) required no further hemorrhoidal treatment (Fig. 1). Persistent symptoms without complete resolution after treatment (treatment failure) were observed in five patients (33.3%) during the 6-month follow-up period. One patient (6.7%) required surgical treatment with a Milligan–Morgan hemorrhoidectomy within 30 days because of persistent symptoms and was therefore not included in the 6-month PROM assessment. Four patients (26.7%) underwent a second session of sclerotherapy at the end of the 6-month follow-up period because of incomplete symptom resolution, achieving complete resolution thereafter. No true recurrences, defined as reappearance of symptoms after complete initial resolution, were observed during the 6-month follow-up. No major complications occurred.

### Preoperative PROMs description

PROMs results are presented in Table 1. At baseline, the mean PROM-HISS score was 13.4 ± 3.0. Among individual PROM-HISS symptom domains, prolapse showed the highest mean score, followed by bleeding, pain, soiling, and itching (Table 1). Pearson correlation analysis demonstrated that the overall HISS score was strongly associated with pain (*r* = 0.800), prolapse (*r* = 0.717), and soiling (*r* = 0.655), while moderate correlations were observed with itching (*r* = 0.585) and bleeding (*r* = 0.408). At baseline, the mean HDSS score was 10.6 ± 3.1. Among individual HDSS symptom-frequency domains, prolapse showed the highest mean value, followed by bleeding, pain, soiling, and itching (Table 1). Pearson correlation analysis demonstrated that the overall HDSS score was most strongly associated with prolapse (*r* = 0.709), followed by soiling (*r* = 0.674) and pain (*r* = 0.647). Moderate correlations were observed with bleeding (*r* = 0.476), whereas itching showed only a weak association with the overall score (*r* = 0.289).

**Table 1:** Patient-reported outcomes (PROMs) at baseline, 7-day, 30-day, and 6-month follow-up. Data are presented as mean ± standard deviation. Statistical analysis for longitudinal comparisons across timepoints was performed using one-way repeated-measures ANOVA. Pairwise post-hoc comparisons were adjusted using the Benjamini–Hochberg false discovery rate correction

	Preop. (*n* = 15)	7 days (*n* = 15)	30 days (*n* = 15)	6 months (*n* = 14)*
Pain HISS	2.8 ± 1.6	1.7 ± 1.2	1.4 ± 1.0	1.8 ± 1.0
Pain HDSS	2.1 ± 1.5	0.7 ± 1.3	0.8 ± 1.2	1.0 ± 0.4
Bleeding HISS	3.2 ± 1.1	1.7 ± 1.1	2.2 ± 1.2	2.2 ± 1.1
Bleeding HDSS	2.8 ± 1.2	0.9 ± 1.4	1.6 ± 0.8	1.2 ± 1.2
Prolapse HISS	3.7 ± 0.9	2.3 ± 0.9	2.1 ± 0.9	2.2 ± 0.9
Prolapse HDSS	3.4 ± 1.0	2.2 ± 1.7	2.2 ± 1.4	2.3 ± 1.5
Soiling HISS	1.9 ± 1.3	1.3 ± 0.9	1.6 ± 1.0	1.4 ± 0.7
Soiling HDSS	1.4 ± 1.2	0.2 ± 0.4	0.6 ± 0.7	0.9 ± 1.5
Itching HISS	1.8 ± 1.2	1.6 ± 1.0	1.2 ± 0.4	1.0 ± 0.2
Itching HDSS	0.9 ± 1.0	0.9 ± 1.1	0.7 ± 0.9	0 ± 0
HISS score	13.4 ± 3.0	8.6 ± 3.4	8.6 ± 2.4	8.6 ± 2.5
HDSS score	10.6 ± 3.1	4.9 ± 3.2	5.9 ± 2.9	5.4 ± 2.1

*One patient underwent Milligan–Morgan hemorrhoidectomy and was therefore not included in the 6-month PROM assessment

### Postoperative PROMs modifications

**Fig. 2:**
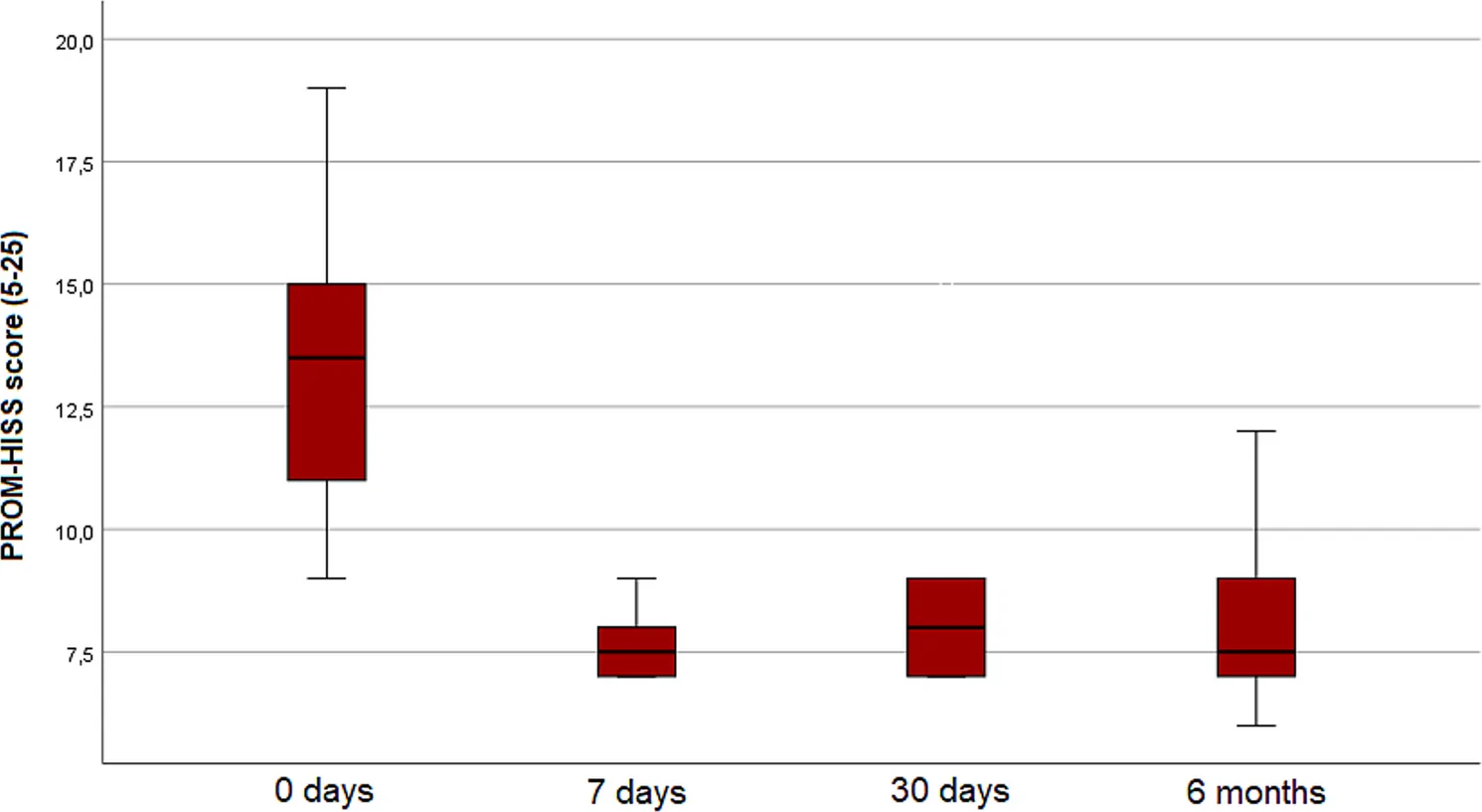
Box plots showing changes in PROM-HISS symptom domain scores over time

**Fig. 3:**
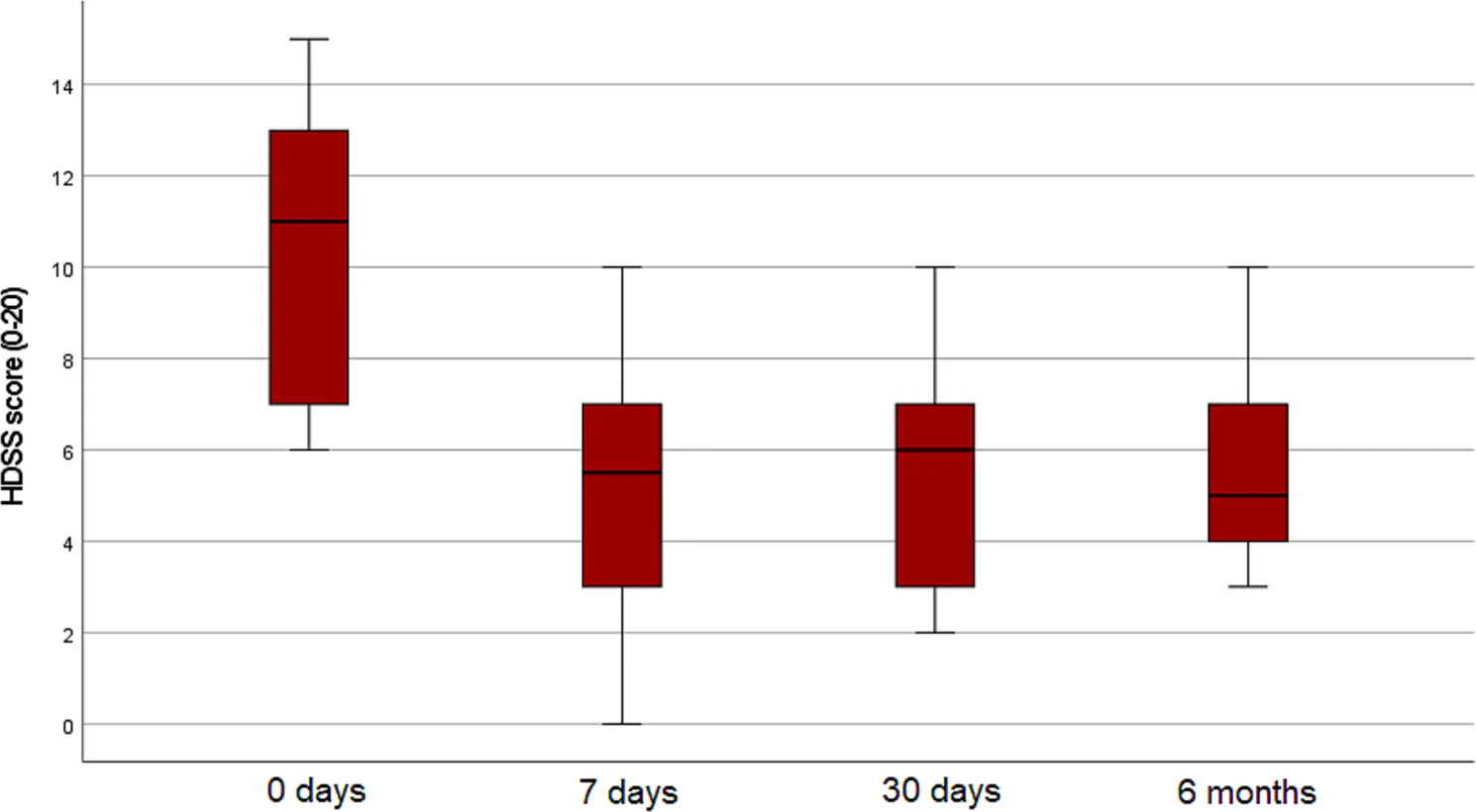
Box plots showing changes in HDSS symptom domain scores over time

**Fig. 4:**
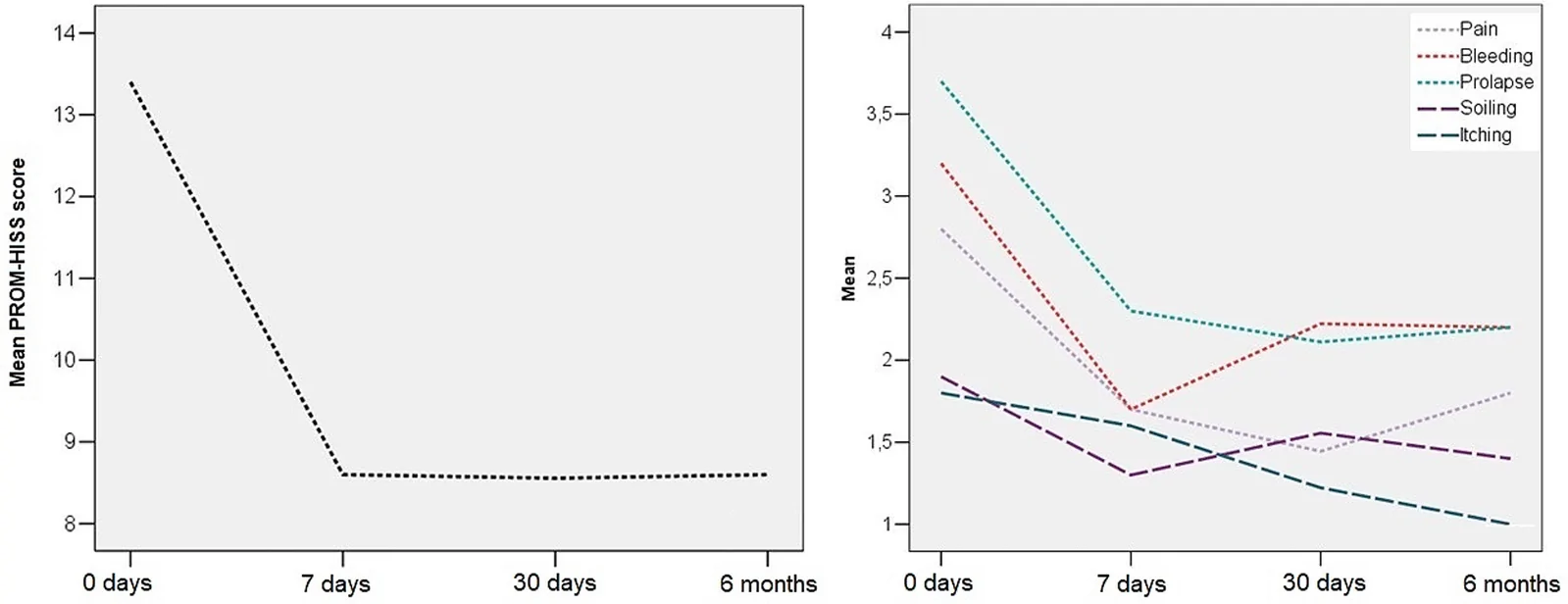
Temporal trends of the PROM-HISS score (left panel) and its individual symptom components (right panel) over time. Mean values are reported at each time point

A significant difference was present between the preoperative and postoperative PROM-HISS and HDSS symptom domains at all timepoints (Figs. 2 and 3). With regards to PROM-HISS, repeated-measures ANOVA demonstrated a significant effect of time for pain, bleeding, and prolapse scores (*p* = 0.005, *p* = 0.001, and *p* = 0.001, respectively), whereas no significant changes were observed for soiling and itching (*p* = 0.178 and *p* = 0.119; Fig. 4). Pain scores significantly decreased from baseline to day 7 (*p* = 0.040), day 30 (*p* = 0.023), and 6 months (*p* = 0.032). Bleeding showed a marked reduction at day 7 (*p* = 0.005) and day 30 (*p* = 0.026), but no significant differences at 6 months compared to baseline levels (*p* = 0.052). Prolapse scores significantly improved at day 7 (*p* = 0.025), day 30 (*p* = 0.002), and 6 months (*p* = 0.012) (Fig. 4). In contrast, soiling and itching did not show statistically significant variations at 7 days (*p* = 0.111, *p* = 0.678 respectively), 30 days (*p* = 0.366, *p* = 0.181 respectively), and 6 months (*p* = 0.213, *p* = 0.070 respectively) (Fig. 4).

**Fig. 5:**
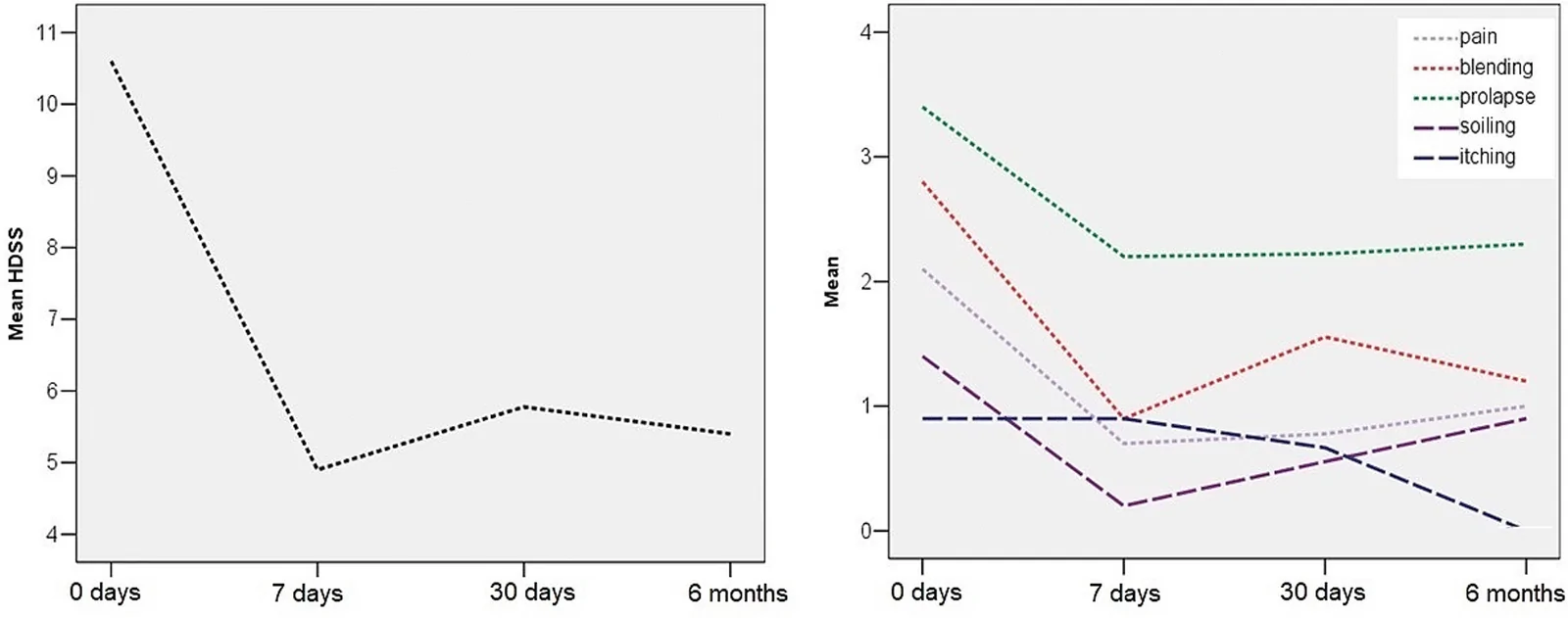
Temporal trends of the HDSS score (left panel) and its individual symptom components (right panel) over time. Mean values are reported at each time point

With regards to HDSS symptom domain, repeated-measures ANOVA showed a significant effect of time on frequency of pain, bleeding, soiling, and itching (*p* = 0.008, *p* = 0.031, *p* = 0.038, and *p* = 0.008, respectively), whereas no significant temporal variation was observed for prolapse (*p* = 0.237; Fig. 5). Pain frequency showed a significant reduction at day 7 compared with baseline (*p* = 0.029) day 30 (*p* = 0.021), and 6 months (*p* = 0.024) (Fig. 5). Bleeding frequency significantly decreased at day 7 (*p *= 0.002), day 30 (*p* = 0.026), and 6 months (*p* = 0.008) (Fig. 5). Soiling frequency showed a significant early reduction at day 7 (*p* = 0.018), followed by no significant difference at 30 days (*p* = 0.061) and 6 months (*p* = 0.244) compared to baseline (Fig. 5). Itching and prolapse showed no significant reduction of frequency compared to baseline at day 7 (*p* = 1.000, *p* = 0.074 respectively), 30 days (*p* = 0.173, *p* = 0.056 respectively), and 6 months (*p* = 0.154, *p* = 0.146 respectively) (Fig. 5).

## Discussion

Recent evidence has renewed interest in sclerotherapy, particularly with the introduction of PF, which offers improved safety and ease of administration compared to older agents. While favorable outcomes have been reported in low- and intermediate-grade HD [3], data in grade IV patients remain extremely limited. In the present study, PF sclerotherapy was associated with a significant improvement in PROMs in a selected cohort of patients with Goligher grade IV HD who refused surgical treatment. In our experience, numerous patients with high Goligher grades specifically requested a less invasive, symptom-focused palliative treatment even after extensive counseling regarding the limitations associated with such approaches in grade IV HD. The longitudinal analysis suggests an early therapeutic effect, most evident within the first 7–30 days, followed by a plateau or partial attenuation of improvement at 6 months, particularly for bleeding-related outcomes. This pattern could be explained by an initial sclerosing and inflammatory obliteration effect with partial recanalization or collateral vascular adaptation over time. Still, two-thirds of such patients requested no further treatment at 6-month follow-up, and the need for hemorrhoidectomy was limited to a single case (6.7%).

Traditionally, grade IV HD has been considered an indication for excisional hemorrhoidectomy due to the high recurrence rates associated with less invasive treatments [9]. However, this paradigm is primarily based on surgeon-centered outcomes, such as anatomical correction and recurrence of prolapse, rather than on patient-perceived symptom burden. While Goligher grading represents a strict anatomical staging system that guides mandatory escalation of care, contemporary evidence has encouraged increasing interest in a symptom-driven therapeutic paradigm, in which treatment selection is guided primarily by symptom burden, functional impairment, and patient preference, rather than anatomical grade alone [15, 16]. Our study specifically focused on the patient’s perspective, suggesting a possible discrepancy between anatomical severity and symptomatic burden. Although not curative, sclerotherapy may provide meaningful symptom control in selected patients with advanced disease not willing to undergo hemorrhoidectomy. In this context, our findings add preliminary observations suggesting that symptom-oriented treatment strategies may have a role even in advanced disease [8], particularly when surgery is declined or contraindicated. Although recent studies have explored the use of PF sclerotherapy in combination with excisional surgery in grade IV HD [17], our findings provide preliminary observations supporting the feasibility of PF sclerotherapy as a standalone, symptom-oriented option. From a practical perspective, the therapeutic dilemma addressed in the present study is not whether PF sclerotherapy should replace hemorrhoidectomy, but rather what treatment may reasonably be offered to patients who continue to decline surgery despite appropriate counseling. Oral flavonoids may provide temporary symptom relief; their efficacy in advanced grade IV disease is generally limited. Moreover, in patients receiving anticoagulant or antiplatelet therapy, persistent bleeding may remain difficult to control with conservative treatment alone, further narrowing the available non-surgical therapeutic options. Within this scenario, PF sclerotherapy should be regarded as a symptom-oriented, palliative strategy rather than a curative alternative to surgery, aiming to improve quality of life while preserving the possibility of future definitive surgical treatment if required.

A key strength of this study lies in the use of PROMs to assess treatment outcomes. The symptom-focused PROM-HISS and HDSS subsets demonstrated significant improvements over time, suggesting a possible association between PF sclerotherapy and reduced symptom burden. Importantly, these tools capture complementary aspects of the disease—symptom intensity and frequency—which are not necessarily aligned in individual patients. A more granular interpretation of domain-specific outcomes revealed a heterogeneous response pattern across symptoms. Pain and bleeding showed the most consistent and sustained improvement over time, whereas soiling and itching remained largely unchanged across follow-up. Prolapse demonstrated an intermediate and more complex behavior, with a significant reduction in severity (HISS domain) but a less consistent effect on frequency (HDSS domain). This divergence suggests that PF sclerotherapy may reduce the subjective burden of prolapse without fully eliminating episodic recurrence, highlighting a clinically relevant distinction between symptom impact and symptom occurrence. The observed discordance between symptom severity and frequency measures also suggests that PROM selection may substantially influence the perceived magnitude of treatment benefit in HD.

This study has several limitations. First, the small sample size and retrospective design limit the generalizability of the findings and reduce the statistical power of the analyses—the limited sample size increases the risk of type II error and reduces the robustness of subgroup and longitudinal analyses; therefore, the results should be interpreted cautiously and considered exploratory, hypothesis-generating rather than practice-changing. Furthermore, the retrospective observational design may have introduced selection, performance, and detection biases that cannot be completely excluded despite the use of predefined institutional follow-up protocols. In addition, the study population consisted exclusively of patients who declined hemorrhoidectomy despite appropriate counseling. Consequently, the findings may not be generalizable to the overall population of patients with Goligher grade IV hemorrhoidal disease and should be interpreted within the context of this highly selected subgroup. Second, the absence of a comparative treatment group prevents direct comparison with other therapeutic approaches, including standard surgical treatment, and also precludes definitive attribution of the observed clinical improvements to PF sclerotherapy alone, as these findings may have been partially influenced by spontaneous symptom fluctuation, regression to the mean, placebo effect, or other unmeasured confounding factors. Third, subsets of the PROM-HISS and HDSS instruments were used rather than the fully validated original versions [12, 13]. This does not question the validity or reproducibility of the original PROM-HISS and HDSS instruments themselves, which remain fully validated. Rather, the exploratory nature of the present analyses derives from the separate use of selected symptom domains outside the context of the complete validated composite instruments. Although the retained symptom domains represent the core symptom constructs originally developed within both instruments, exclusion of the daily activity, treatment satisfaction, and quality-of-life domains may have reduced the ability of the scores to capture the broader patient experience and overall disease burden. Furthermore, these symptom-focused subsets have not undergone independent psychometric validation. Consequently, the observed changes should be interpreted as exploratory assessments of symptom severity and frequency rather than definitive evaluations based on the fully validated PROM-HISS and HDSS/SHD-HD instruments. Future adequately powered prospective studies using the complete validated PROM-HISS and HDSS/SHD-HD instruments are required to confirm these preliminary observations. Finally, although patients were followed for 6 months, this duration may still be insufficient to adequately assess long-term recurrence and durability of symptom control in advanced hemorrhoidal disease. Longer-term evaluation is required to better define durability of symptom control after PF sclerotherapy.

## Conclusions

This study reflects real-world clinical practice and addresses an important unmet need in a subset of patients who are often underrepresented in clinical trials. Overall, these preliminary exploratory findings suggest that selected patients with Goligher grade IV hemorrhoidal disease who decline hemorrhoidectomy may obtain short-term symptom relief following PF sclerotherapy. Sclerotherapy should not be considered a substitute for hemorrhoidectomy in patients seeking definitive anatomical correction; however, in carefully selected patients with Goligher grade IV HD who are unwilling or unfit to undergo surgery, it may be considered a feasible symptom-oriented treatment approach within the context of individualized clinical decision-making. Future adequately powered prospective comparative studies should aim to validate these findings in larger prospective cohorts, incorporate standardized composite symptom and quality-of-life scores, and better delineate clinical and functional predictors of response, in order to identify patients who may derive the greatest benefit from a symptom-oriented, minimally invasive strategy.

## Data Availability

Database are available from the Authors upon reasonable request.
